# Plant-Derived Terpenoids: A Plethora of Bioactive Compounds with Several Health Functions and Industrial Applications—A Comprehensive Overview

**DOI:** 10.3390/molecules29163861

**Published:** 2024-08-15

**Authors:** José S. Câmara, Rosa Perestrelo, Rui Ferreira, Cristina V. Berenguer, Jorge A. M. Pereira, Paula C. Castilho

**Affiliations:** 1 CQM—Centro de Química da Madeira, NPRG, Universidade da Madeira, Campus Universitário da Penteada, 9020-105 Funchal, Portugal; rmp@staff.uma.pt (R.P.); rui.ferreira@staff.uma.pt (R.F.); cristina.berenguer@staff.uma.pt (C.V.B.); jorge.pereira@staff.uma.pt (J.A.M.P.); pcastilho@staff.uma.pt (P.C.C.); 2 Departamento de Química, Faculdade de Ciências Exatas e da Engenharia da Universidade da Madeira, Campus Universitário da Penteada, 9020-105 Funchal, Portugal

**Keywords:** terpenoids, biosynthesis, biosources, bioactive compounds, health benefits, industrial applications

## Abstract

Terpenoids are a large class of natural secondary plant metabolites which are highly diverse in structure, formed from isoprene units (C-5), associated with a wide range of biological properties, including antioxidant, antimicrobial, anti-inflammatory, antiallergic, anticancer, antimetastatic, antiangiogenesis, and apoptosis induction, and are considered for potential application in the food, cosmetics, pharmaceutical, and medical industries. In plants, terpenoids exert a variety of basic functions in growth and development. This review gives an overview, highlighting the current knowledge of terpenoids and recent advances in our understanding of the organization, regulation, and diversification of core and specialized terpenoid metabolic pathways and addressing the most important functions of volatile and non-volatile specialized terpenoid metabolites in plants. A comprehensive description of different aspects of plant-derived terpenoids as a sustainable source of bioactive compounds, their biosynthetic pathway, the several biological properties attributed to these secondary metabolites associated with health-promoting effects, and their potential industrial applications in several fields will be provided, and emerging and green extraction methods will also be discussed. In addition, future research perspectives within this framework will be highlighted. Literature selection was carried out using the National Library of Medicine, PubMed, and international reference data for the period from 2010 to 2024 using the keyword “terpenoids”. A total of 177,633 published papers were found, of which 196 original and review papers were included in this review according to the criteria of their scientific reliability, their completeness, and their relevance to the theme considered.

## 1. Introduction

**Figure 1:**
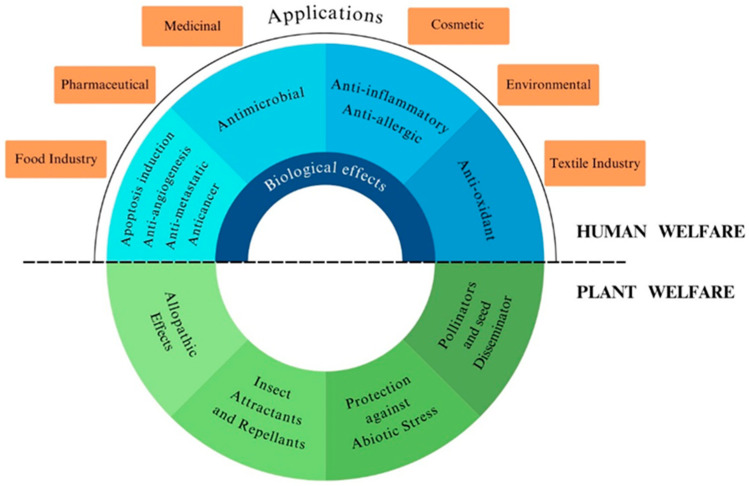
Importance of terpenoids in plant and human welfare and potential industrial fields of application.

Terpenoids, found in almost all classes of living organisms, represent the largest group of natural compounds and have an extraordinary structural diversity, being derived from carbon backbones of 2-methylbuta-1,3-diene (isoprene units, C5) rearranged into linear and cyclic structures. They are classified as monoterpenes (2 C-5 units), sesquiterpenes (3 C-5 units), diterpenes (4 C-5 units), triterpenes (6 C-5 units), and so forth, and are categorized into six fundamental classes: tocopherols, taxanes, ingenanes, artemisinins, sterols, and cannabinoids [1]. Some of them have been shown to possess a wide range of biological activities, including antioxidant, anti-inflammatory, antimicrobial, anticancer, and antiallergic properties (Figure 1) [1]. They have huge economic value as well as a wide range of applications in different fields, including the food, pharmaceutical, cosmeceutical, and chemical industries, in addition to their environmental value (Figure 1).

### Biosynthetic Pathways

It is now accepted that two mechanisms, the mevalonate pathway (MVA) and the methylerythritol phosphate (MEP) pathway, together with glycolysis, convert glucose into the two five-carbon atoms, universal precursors of terpenoids: dimethylallyl diphosphate (DMAPP) and isopentenyl diphosphate (IPP). Both pathways involve an 18-enzyme cascade [2], which can be summarized via the following steps: activation (enzymes 1 and 2 activate the precursor molecule, often through phosphorylation or isomerization); intermediate formation (subsequent enzymes 3–6 modify the precursor by adding or removing functional groups, leading to the formation of key intermediates); cyclization and structural modification (enzymes 7–8 catalyze cyclization and introduce structural changes, refining the molecule’s core structure); oxidation and reduction (enzymes 9–11 carry out oxidation and reduction reactions, further modifying the intermediate to approach the final product); stereochemical adjustments (enzymes 13–16 alter the stereochemistry to ensure the correct 3D orientation of the molecule); and final modifications (enzymes 17 and 18 add groups like acetyl or sugar moieties, stabilize the product, and prepare it for release).

For the MVA pathway, the starting primary metabolite, the acetyl-CoA, is converted to acetyl-SCoA, which goes through a series of Claisen reactions to condense three molecules of acetyl-SCoA to mevalonic acid. Claisen reactions result in a C-C bond between a single ester and one carbonyl compound, and in this case, one acetyl-CoA molecule serves as an electrophile at C-1 and the other as the equivalent of a C-2 carbanion. Both hydroxyl groups of the mevalonic acid undergo phosphorylation followed by decarboxylation with elimination of a phosphate group, which directly generates IPP and its isomer DMAPP. Both IPP and DMAPP can also be formed through the methylerythritol phosphate (MEP) pathway in plastids, including the chloroplast in plants, using the pyruvate and glyceraldehyde-3-phosphate from glycolysis to produce deoxyxylulose-5 phosphate, which is reduced to MEP. A series of reactions convert MEP into a mixture of IPP and DMAPP. This pathway is of special relevance to bacteria [3,4]. While C5 units derived from the MVA pathway are used in the formation of compounds such as sesquiterpenes, sterols, and triterpenes, C5 units of the MEP pathway are used in the production of isoprene, monoterpenes, diterpenes, chlorophylls, and carotenoids.

**Figure 2:**
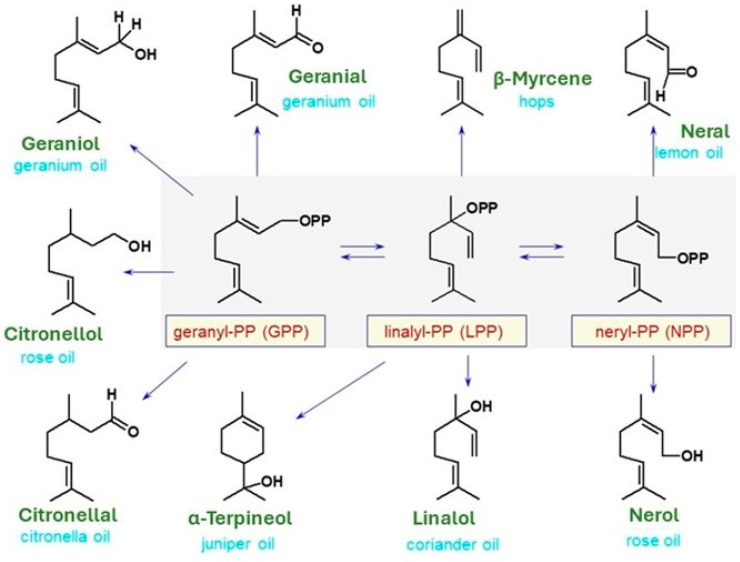
The isomerization of GPP to nerylPP and the rearrangement to linalylPP opens the possibility for a cyclic carbocation (terpinyl cation) by positioning an allyl electron-deficient carbon close to the nucleophilic double bond.

The condensation of DMAPP and IPP in head-to-tail mode (nucleophilic attack of IPP’s terminal double bond on the especially reactive allylic carbon of DMAPP) provides a 10-carbon skeleton of geranyl diphosphate (GPP), which is the immediate precursor of all monoterpenes. A further IPP addition to GPP’s allylic carbon leads to the formation of farnesyl pyrophosphate (FPP), which is the precursor of sesquiterpenes (15 carbons) and another IPP addition yields geranylgeranyldiphosphate (GGPP) as a precursor of diterpenes (20 carbon). All these additions are “head-to-tail”, connecting C1 from one unit to C4 of the next unit. Triterpenes (30 carbons) and tetraterpenes (40 carbons) result from the dimerization of FPP and GGPP, respectively, in a “tail-to-tail” fashion condensation. Monoterpenoids and sesquiterpenoids show an enormous variety of structures due to the instability of the carbocations resulting from the removal of the terminal diphosphate group from GPP and FPP, respectively. Due to cyclase enzymes, which catalyze the formation of a cyclic compound, GPP can undergo isomerization to nerylPP or rearrangement to linalylPP, from which cyclic structures may occur (Figure 2).

**Figure 3:**
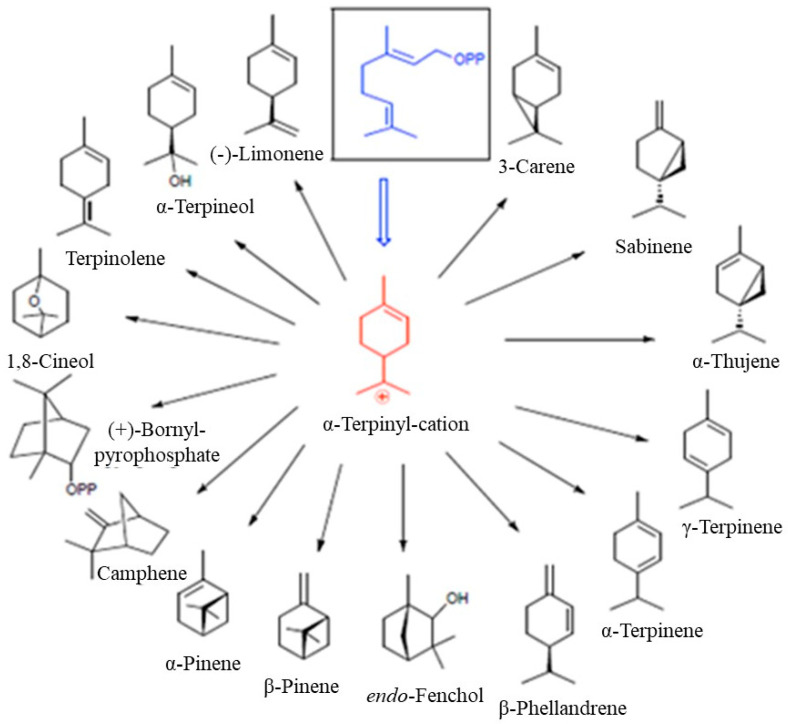
Structural diversity of monoterpenes.

Monoterpenocyclase enzymes use GPP as a substrate and typically catalyze a single cyclization reaction. However, sometimes, you look at more than one product. These enzymes require Mg^2+^ or Mn^2+^ as a cofactor. The resulting α-terpinyl cation remains bound to the enzyme in many cyclase-catalyzed transformations and can then react in many ways since several types of rearrangements, such as 1,2- or 1,3-hydrogen or alkyl migration, are possible. Further enzyme-catalyzed reactions, including cyclization, hydroxylation, dehydrogenation, oxidation and/or reduction, isomerization, and conjugation, lead to a myriad of possible 10-carbon (2 × C5 blocks) natural compounds (Figure 3).

**Figure 4:**
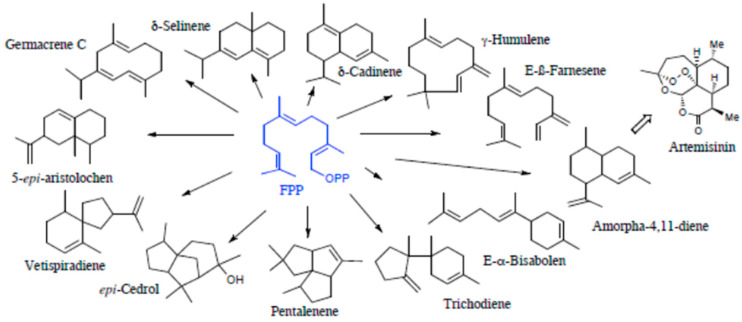
The diversity of structures of sesquiterpenes.

Being 15-carbon skeleton compounds of C5 building blocks, the sesquiterpenes can lead to an even more diverse group of natural products, including some acyclic structures such as farnesene, farnesol, and nerolidol, and a vast array of derivatives from distinct monocyclic, bicyclic, and tricyclic structures (Figure 4). They are biosynthesized by sesquiterpene synthase.

Farnesyl pyrophosphate (FPP) is the precursor to sesquiterpenes. It can hydrolyze farnesol or rearrange nerodyl pyrophosphate, which is the substrate of several enzymes in the cyclase family, giving rise to monocyclic sesquiterpenes, such as humulene, longifolene, germacrene, and bisabolene. Bi- and tricyclic chain structures and polycyclic spiro structures are common. There are more than 200 different cyclic structures. The different sesquiterpene carbon skeletons are formed through irregular coupling reactions. Cyclization is initiated by the metal-ion-induced ionization of pyrophosphate (PP) to form allyl cations, facilitating the structural shift and catalyzing cyclization closure. FPP undergoes one or more cyclizations to form intermediates, which are then converted to sesquiterpene skeletal end-products under the action of the synthase enzyme [5].

The reaction mechanism can be divided into four groups, two involving (2*E*,6*E*)-FPP and two resulting from a previous isomerization of (2*E*,6*E*)-FPP to (3*R*)-nerolidyl diphosphate ((3*R*)-NPP) [6]. Depending on the sesquiterpene synthase, (2*E*,6*E*)-FPP ionization and cyclization form either a 10-membered ring of the carbon-positive intermediate, the *E,E*-germacradienyl cation, through a 1,10-cyclization reaction, or the 11-membered ring of the carbocation intermediate, *trans*-humulyl cation, through a 1,11-cyclization reaction. In the second group, ((3*R*)-NPP) can form a 10-membered ring of the carbon-positive intermediate *Z*,*E*-germacradienyl cation through 1,10 cyclization and can form a 6-membered ring of the carbocation intermediate (6*R*)-β-bisabolol cation through a 1,6 cyclization reaction [6,7].

This paper aims to provide a comprehensive review of different aspects of plant-derived terpenoids as a sustainable source of bioactive compounds, their biosynthetic pathway, the several biological properties attributed to these secondary metabolites associated with health-promoting effects, the emerging extraction methods, and their potential industrial applications in several fields. This paper provides an in-depth and comprehensive exploration of terpenoids, highlighting their natural occurrence in plants, metabolic pathways, functions within plants, potential industrial applications, health benefits, and the emerging extraction methods. In addition, future research perspectives in this framework will be highlighted. Terpenoids are discussed for their diverse biological properties and their significance in various industries.

## 2. Terpenoids as Semiochemicals

### 2.1. Insect–Plant Activity

Terpenoids are a diverse class of organic compounds of low molecular weight (300 Da), synthesized as part of the plant secondary metabolism, which includes hemi-, mono-, homo-, sesqui-, and some diterpenoids, and play a significant role in mediating interactions between plants and insects [8]. Secondary metabolism refers to the set of metabolic pathways that produce compounds not directly involved in the normal growth, development, or reproduction of an organism. These compounds often serve ecological functions, such as defense or signaling. Based on these interactions, the emissions of volatile organic compounds, mainly consisting of terpenoids, play an important role as signals between plants and the surrounding environment, including neighboring plants, pollinators and seed dispersers, predators, parasitoids, and herbivores [9]. The characteristic of the volatile mixture is subject to the identity and relative proportion of the individual components and their efficacy depends on the sensitivity of the herbivores and predators [10]. Understanding the mechanisms and spectrum of action of terpenoids on insect targets is crucial for the application of nature-derived by-products in the development of new (bio)insecticides [11].

#### 2.1.1. Terpenoids with Toxic or Repellent Properties 

Many plants produce terpenoids as a chemical defense mechanism against herbivorous insects via the synthesis and emission of volatile organic compounds that can directly affect herbivores’ physiology and behavior due to their toxic, repelling, or deterring properties [12].

Thymol, eugenol, caryophyllene oxide, β-citronellol, and α-pinene are widely utilized as insect repellents, mainly against mosquitoes, ticks, and termites [11,13]. Moreover, several studies demonstrate that terpenoids also act as neurotoxic compounds, causing hyperactivity in the first step and eventually death [11]. Pavela [14] has provided evidence that some essential oils (EOs) components, mostly terpenoids, when applied in binary mixtures, can exhibit synergistic or antagonist effects, with 435 binary combinations tested, of which 135 combinations showed a significant synergic effect, while 150 combinations showed a significant antagonistic effect on the mortality of third instar larvae of *S. littoralis*, an important polyphagous pest.

The inhibition of acetylcholinesterase (AChE), an enzyme involved in neuro–neuronal and neuromuscular junctions in both insects and mammals, is one of the most investigated mechanisms of action of terpenoids. Research shows that some of the terpenoids, such as α and β pinene, carvacrol, and limonene, present in EO, act as competitive inhibitors (i.e., compete they with the substrate for binding to an active site, usually caused by substances that are structurally related to the substrate) and others as uncompetitive inhibitors (i.e., molecules that reversibly bind to an enzyme–substrate complex, yielding an inactive complex) [15]. Such competitive inhibitors attach to the active sites in AChE and prevent the binding of the neurotransmitter acetylcholine (ACh), causing a decrease in the binding of the neurotransmitter but not influencing the maximal activity of the enzyme. On the other hand, the uncompetitive inhibitors bind to the enzyme–substrate complex rather than to the enzyme itself and allosterically alter the action of the enzyme and thus prevent product formation [11]. Anderson et al. [16] determined that the inhibition of AChE requires mM concentrations of EOs, while, usually, the neurotoxic symptoms of EOs are visible at concentrations which are smaller by three orders of magnitude.

Another proposed mechanism of terpenoid action is a positive allosteric modulation (i.e., binding to an enzyme or receptor at a site other than the active site, enhancing its activity) of gamma-aminobutyric acid receptors (GABArs). GABA is the major inhibitory neurotransmitter in the nervous system and the muscles in both mammals and insects [17]. Several studies demonstrate the potentiation of GABA’s effect on mammalian receptors induced by EO, with menthol, thymol, and other monoterpenoids causing strong potentiation of the Cl^−^ current induced by the GABA neurotransmitter, even with low µM concentrations [11]. 

The insect GABArs are related to vertebrate ionotropic GABArs and, as such, mediate the inhibitory effect on neurotransmission in the insect nervous system [11]. The insect GABArs are targets for several chemical insecticides that act as antagonists of the GABArs and induce inhibition or overexcitation of the insect nervous system [11]. Diverse studies evaluated the efficacy of EOs for their insecticide activities [18], where it was observed that monoterpenoids such as thymol, carvacrol, and pulegone increased the GABA-induced Cl^−^ uptake in the insect membrane. It was proposed that these EO components modulate the insect GABA system by binding at the receptor and increasing chloride anion influx into the neurons, effectively behaving as positive allosteric modulators of the insect GABA receptors [11].

Terpenoids also act via the insect octopaminergic system. Octopamine (OA) acts as a neurotransmission, neuromodulator, and neurohormone, and plays an important role in the regulation of insect activity, stress response, and social behavior [11]. This molecule binds to specific G protein-coupled membrane receptors (OAr), activating the enzyme adenyl cyclase that mediates either increases or decreases in the intracellular signaling molecule cyclic AMP (cAMP) and levels of intracellular Ca^2+^, leading to activation of the calcium-dependent protein kinase C (PKC) [19]. Available studies demonstrate that EO components, and terpenoids in particular, interact with OAr and tyramine (TA) receptors by competing with OA in binding and acting as agonists of these receptors [20]. Low concentrations (µM) of monoterpenes such as thymol, carvacrol, eugenol, and α-terpineol cause an increase in both the cAMP level and the intracellular Ca^2+^ level and induce the activation of kinases PKA and PKC and the phosphorylation of ion channels, enzymes, and receptors [21].

#### 2.1.2. Terpenoids as Attractants

In some cases, plants produce terpenoids to attract specific insects that play a role in their pollination or seed dispersal. A study by Chen et al. [22] on the relationship between fig (*Ficus hispida*) and its pollinator, the *Ceratosolen solmsi marchali* wasp, concluded that only the female floral stage is receptive to its pollinator and that, in the next stage of figs development, the pollinator response diminished (Table 1). The insect is responsive to both changes in concentration and quality. The volatiles present during the development stage were linalool, limonene, and β-pinene, with the presence of different isomers during the two stages studied. Another study reveals that terpene compounds can be finely adjusted to a certain concentration to repel or attract pollinators, with the volatile compounds’ emission being dependent on the daily thermogenic phase, increasing with the increase in temperature [8].

An effective indirect defense against herbivores consists of the emission of volatile terpenes and terpenoids that attract the parasitoids of specific herbivores. The monoterpene 1,8-cineole, present in the EO of cultivars of *Brassica oleracea*, apart from their known insecticidal properties, has the characteristic of attracting the parasitoid *Cotesia glomerata*, which lay eggs in the larvae stage of the butterfly *Pieris brassicae*, which cause extensive damage to cabbage cultures [23].

It has been observed that some terpenoids act as chemo-attractants to herbivores. In a study, different constitutive and induced terpenes (γ-terpinene and α-pinene) were synthesized in increasing concentrations on *Eucalyptus grandis* in response to attacks from eucalyptus gall wasp *Leptocybe invasa*, acting as pest attractants; whereas iso-pinocarveol, also produced, acted to recruit parasitoids for this wasp [24]. Also, the cultivation of transgenic plants overexpressing attractants may effectively lure herbivores into entrapments that prevent escape [11].

**Table 1:** Production of terpenes in plants and their targets and effects against insects.

Plant Species	Terpene ID and Class	Target	Mechanism and Effect	Ref.
**Dalechampia** (*Euphorbiaceae*)	Oxygenated terpenoid resins	Female euglossine *(Aphidae*), female anthidiine (*Megachilidae*) bees	Reward to pollinators	[25,26]
**Fig** (*Ficus hispida*)	Linalool; limonene and β-pinene (monoterpenes)	Wasp (*Ceratosolen solmsi marchali*)	Signaling pollinators	[27]
**Sweet rocket** (*Hesperis matronalis*)	Linalool; β-ocimene (monoterpenes)	Mainly syrphid flies (*Syrphidade*)	Attractant to pollinators	[28]
**Cabbage** (*Brassica species*)	1,8-cineole (monoterpenes)	Parasitic wasps (*Cotesia glomerata*)	Attractant to parasitoids that lay eggs in herbivores larvae	[23]
**Various plant species**	(*Z*)-3-hexenyl acetate; (*Z*)-3-hexenol; (3*E*)-4,8-dimethyl-1,3,7-nonatriene; and linalool	Pest predator (*Chrysopa phyllochroma*)	Attractant to predators and promotes oviposition	[29]
**Tomato** (*Solanum lycopersicum*) and **tobacco** (*Nicotiana tabacum*)	Β-ocimene (monoterpenes)	Parasitoid (*Aphidius ervi*) Pest (*Macrosiphum euphorbiae*)	Attractant to parasitoids and defense against pest	[30]
**Lavender** (*Lavandula angustifolia*)	Β-*trans*-ocimene; (+)-*R*-limonene (monoterpenes)	Aphids (*Aphidoidea family*)	Pest deterrent	[31]
**Cinnamon** (*Cinnamomum* genus) and **Clove** (*Syzygium aromaticum*)	Eugenol; caryophyllene oxide; α-pinene; α-humulene and α-phellandrene (monoterpenes)	Wheat weevil (*Sitophilus granaries*)	Toxic and repellent effects on adult specimens	[32]
**Eucalyptus** (*Eucalyptus grandis*)	A-pinene; γ-terpinene (monoterpenes)	Eucalyptus gall wasp (*Leptocybe invasa*)	Attractant to pest	[24]
**Various plant species**	Geraniol (terpenoid)	Sweet potato whitefly (*Bemisia tabaci*)	Encapsulated geraniol shows attraction to that specimen	[33]

### 2.2. Antifungal Activity

Terpenoid compounds are often produced by plants as a defense mechanism against various pathogens, including fungi. The antifungal activity of terpenes and terpenoids might be caused by their highly lipophilic nature and low molecular weight, causing cell death or inhibiting the sporulation and germination of food spoilage fungi [34].

The capacity of disruption of the cell membrane by thymol and its closely related isomer carvacrol, by interacting with vesicles and cell membranes and through impaired ergosterol biosynthesis in Candida strains, leads to cell membrane integrity being affected by osmotic and metabolic instability and is one of the most well-known antifungal mechanisms of action [35]. Also, the synthesis of β-glucan and chitin, glucose polysaccharides that form the backbone of the cell wall, can be inhibited by limonene and thymoquinone, leading to damage in the fungal cell wall [36].

Mitochondrial effectiveness could be impaired by terpenes, inhibiting the action of mitochondrial dehydrogenases involved in ATP biosynthesis [35]. This inhibition can occur via the inhibition of the proton pumps in the respiratory chain, leading to a reduction in ATP production. Haque et al. [37] studied the potential of a triterpenoid and tetraterpenoid in *Saccharomyces cerevisiae* as an antifungal agent and concluded that terpenoids could play a key role in diminishing the mitochondrial content, which gives rise to an altered level of reactive oxygen species (ROS) and ATP generation.

The inhibition of efflux pumps, namely, the fungal plasma membrane H^+^-ATPase, and their role in supporting the large transmembrane electrochemical proton gradient across the cell membrane and regulating intracellular pH, could be impaired by thymol, eugenol, and carvacrol, leading to intracellular acidification and cell death [38]. Also, a study demonstrates that terpenoids such as eucalyptal D display prominent synergistic effects when used in combination with the commonly prescribed antifungal drug fluconazole in resistant strains of Candida spp. via their simultaneous effect on the upregulation of the efflux pump genes CDR1 and MDR1 [39].

The inhibition of fungi nitric oxide synthases (NOS) activity may increase the effectiveness of fungicidal therapy since NO increases resistance to a broad spectrum of antifungal drugs [34]. EOs can reduce the level of nitric oxide and limit H_2_O_2_ production and NO synthase, demonstrating a potential in the therapy of oxidative damage. The isoprenoid farnesol produces a toxic effect on the phytopathogenic fungus *Botrytis cinerea*, suppressing the mycelial growth and triggering apoptosis due to reactive oxygen species (ROS) accumulation [40].

Biofilms are sessile microbial and fungal communities that are strongly attached to surfaces and each other, being protected by a polymeric extracellular matrix (ECM) constituted primarily of polysaccharides. This improves their resistance against the external environment and the application of antifungal drugs and is an important virulence factor for pathogenic fungi [41]. The antibiofilm activity of terpenoids is the object of new natural therapeutic alternatives, mostly derived from increasing resistance to conventional drugs, as well as the possibility of these drugs having undesirable effects on human health [34]. He et al. [42] demonstrated that eugenol displayed potent activity against *C. albicans* biofilms in vitro, with low cytotoxicity, and therefore has potential therapeutic implications for biofilm-associated candidal infections. Another study reported the efficient antibiofilm activity of *Cymbopogon citratus* and *Syzygium aromaticum* against *Candida albicans* biofilms, even being more effective against adaptive mechanisms of resistance to amphotericin B and fluconazole [43].

## 3. Terpenoids iR1n Human Health

Terpenoids have been associated with a wide range of biological activities that are essential to human health. These include neuroprotective effects, preventing neurodegenerative conditions [44] and slowing ageing [45]; antimicrobial activities against both antibiotic-susceptible and antibiotic-resistant bacteria [46]; and involvement in suppressing the microglia-mediated inflammation involved in acute or chronic inflammatory diseases [46]. Some terpenoids, such as azadirachtin, carvone, menthol, ascaridol, methyl eugenol, toosendanin, and volkensin, have been shown to yield antimicrobial effects [46]. Others, such as myrcene, possess anti-inflammatory, analgesic, and antibiotic activities [47]. Additionally, several terpenoids are biologically active and are being exploited in the fight against cancer, malaria, inflammation, and a variety of infectious diseases [48]. However, it is important to note that terpenoids can also be toxic if ingested in large amounts. They are local irritants and can cause gastrointestinal symptoms, altered mental status, seizures, and even coma if ingested in significant amounts [48]. Overall, terpenoids’ effects on human health, both positive and negative, are far from fully understood, and more research is necessary to clarify the benefits and risks of using terpenoids as supplements or for medicinal purposes. Nevertheless, when terpenoids are used in combinations, they can exhibit synergies or interactions that enhance their overall effects. These interactions can be pharmacodynamic or pharmacokinetic. An example of pharmacodynamic synergies is the combination of curcumin and piperine. Curcumin has anti-inflammatory properties, while piperine enhances curcumin’s bioavailability by inhibiting enzymes that metabolize curcumin too quickly. The combination of these terpenoids amplifies the anti-inflammatory effects more than either compound alone. Another example is the combination of terpenoids like menthol and eucalyptol. Menthol acts as a cooling agent and enhances the penetration of eucalyptol, which has anti-inflammatory properties, leading to more effective pain relief. In sum, terpenoids can interact beneficially when used in combination, leading to enhanced efficacy, improved bioavailability, or reduced side effects, making them valuable in both traditional and modern therapeutic approaches.

### 3.1. Terpenoids on Cancer Prevention

Plant-derived terpenoids have proven effective against many different types of cancer cells, both in in vitro and in vivo models [49,50]. Tumorigenesis is a multifaceted process, and its progression is associated with several hallmarks, including uncontrolled cell growth, dysregulation of apoptosis, activation of invasion, induction of angiogenesis, and metastasis [51]. Terpenes have been shown to exert antitumorigenic effects against such processes, suggesting their potential use as chemotherapeutic agents for treating tumors [52]. Table 2 briefly presents recent examples of the protective effects of terpenoids against different forms of cancer.

Overall, terpenoids can act on different mechanisms of cancer progression, notably apoptosis, the cell cycle, ROS production, autophagy, and necroptosis. Among these, sesquiterpene lactones are involved in apoptosis induction, tumor redifferentiation, and inhibition of the post-translational isoprenylation of cell-growth-regulating proteins [79]. Helenalin, for instance, was shown to be very efficient in promoting rhabdomyosarcoma (RMS) cell death in vitro. RMS is the most frequent soft tissue sarcoma in pediatric patients, and the mechanism of helenalin therapeutic effects involves ROS generation, inhibition of NF-κB p65 expression, autophagy induction of enzymatic markers, i.e., Atg12 and LC3-B, and triggering the cleavage of Caspase 3 and 9 [65]. Limonene has been extensively assayed in cancer cells, exhibiting cytotoxicity in gastric cancer, hepatocellular carcinoma, colon cancer, and pancreatic cancer cells [80]. Recently, Alipanah et al. [81] identified limonene as the domain component of several citrus fruits and improved its cytotoxicity against melanoma and breast cancer cells through nanofunctionalization with the natural polymer chitosan. Borneol is another promising terpene with anticancer activity. It is one of the many terpenes found within the cannabis plant, ginger, camphor, thyme, and rosemary and is effective against different forms of cancer, such as glioma [55], esophageal squamous cell carcinoma [56], and melanoma [57,58]. Cucurbitacins are tetracyclic terpenes isolated from plants of the Cucurbitaceae family, such as pumpkins, gourds, and cucumbers, and are effective against cancer. Wu et al. [63] provided evidence that a low-dose cucurbitacin C treatment induced cell cycle arrest at the G1 or G2/M stage in different cancer lines, whereas high-dose treatment of this terpene caused apoptosis in cancer cells. These results were further supported by the observation that a 0.1 mg/kg body weight effectively inhibited the growth of cancer-cell-derived xenograft tumors in athymic nude mice and caused significant apoptosis. In turn, cucurbitacin D was shown to enhance the therapeutic efficacy of docetaxel against prostate cancer, while a similar effect was observed for cucurbitacin E regarding doxorubicin cytotoxicity against NCI-N87 gastric cancer cells [64].

### 3.2. Terpenoids in the Prevention of Cardiovascular Diseases

Terpenoids have been reported to possess potential therapeutic applications in the treatment of cardiovascular disease (CVD) by alleviating cardiovascular-related complications, such as high blood pressure, atherosclerosis, and impaired heart function, by modulating inflammation and apoptosis via the MAPK pathway [82]. Research has also shown that terpenoids can act as natural inhibitors of NF-κB, which is an important factor in controlling the inflammatory process associated with CVD [83]. Table 3 describes the most recent studies (since 2018) involving terpenoids for CVD protection.

Two different mechanisms are currently known to play pivotal roles in the development of CVD, namely, NF-κB and NLRP3. The NF-κB pathway controls the expression of genes involved in inflammation, immune response, and cell survival. Terpenoids like oridonin inhibit the activation of NF-κB by preventing the degradation of IκBα, an inhibitory protein that normally binds to NF-κB. This suppression reduces the production of pro-inflammatory cytokines like TNF-α, IL-6, and IL-1β, leading to decreased inflammation. NLRP3 inflammasome is a multi-protein complex that activates caspase-1, leading to the maturation and secretion of inflammatory cytokines IL-1β and IL-18. Terpenoids such as artemisinin and β-caryophyllene inhibit the activation of the NLRP3 inflammasome by reducing reactive oxygen species (ROS) production, blocking potassium efflux, or directly interfering with the assembly of the inflammasome complex. This modulation can help prevent excessive inflammation associated with various chronic inflammatory diseases. One of them is chronic inflammation, or inflammatory infiltration, triggered by the existence of excessive oxidative damage to the cells, with the NLRP3 being inflammasome widely associated with myocardial infarction, myocardial ischemia–reperfusion damage, heart failure, atrial fibrillation, and hypertension [118]. Another mechanism is programmed cell death, known as apoptosis, which can also contribute to the development of CVD due to the loss of cardiomyocytes [82]. As shown in Table 3, terpenoid compounds are potent antioxidants with anti-inflammatory and antiapoptotic properties in the cardiovascular system. In this context, the NLRP3 inflammasome constitutes an important immune-inflammatory target in the pathogenesis and treatment of CVDs [119] because no clinical NLRP3 inflammasome inhibitor is currently available. Therefore, terpenoids, which have many anti-inflammatory effects, including modulation of the NLRP3 inflammatory pathway [118] (Table 3), are good candidates. However, terpenoids display some properties, such as low solubility and bioavailability, reduced cell penetration, and high instability related to their high volatility, which must be modulated to improve their efficacy in the prevention of CVDs [83]. 

### 3.3. Neuroprotective Effects of Terpenoids

Inflammation is a hallmark of several deleterious effects on cells, including those of the nervous system. Therefore, terpenoids have been widely associated with neuroprotection, although the exact mechanisms involved are still poorly understood. Table 4 lists the terpenoids reported in the literature published over the last five years eliciting neuroprotective effects. The PI3K/Akt signaling pathway enables cells to survive and grow in response to external signals. This pathway is tightly controlled by multiple mechanisms, including cross-talk with other signaling pathways, and its deregulation is associated with several diseases, including neurological conditions. Therefore, the neuroprotective potential of terpenoids has been widely investigated. Montenegro et al. [120], for instance, decided to perform a systematic analysis of the neuroprotective activity of olive oil by-products given content terpenoids content, which included monoterpenes (C10), sesquiterpenes (C15), diterpenes (C20), and triterpenes (C30). This study shows that terpenoids from olive leaf extracts have neuroprotective potential [120]. In another exhaustive study, Xu et al. [121] showed that a list of 33 monoterpenes, including carvacrol, citronellol, eucalyptol (1,8-cineole), (−)-linalool, (+)-linalool, menthol, myrtenal, myrtenol, rotundifolone (piperitenone oxide), sobrerol, thymol, α-limonene, α-terpinen-4-ol, α-terpineol, *p*-cymene, and perillyl alcohol, are promising agents for the prevention or treatment of neurological disorders. In addition to these large-scale studies, research has focused on individual compounds to study their neuroprotective potential. Accordingly, terpenoids such as celastrol, ginsenosides, oleanolic acid, ursolic acid, asiatic acid, erythrodiol, and some triterpenoid saponins have been reported to protect against neuroinflammation and oxidative stress [122]. Table 4 presents more detailed data reported in the literature over the last five years regarding the neuroprotective potential of different terpenoids.

Terpenoids have been found to act on multiple signaling pathways involved in neuroprotection, and their ability to target multiple mechanisms simultaneously makes them promising multi-target agents [123]. As previously mentioned, oxidative damage to cells triggers multiple deleterious processes, leading to different diseases, and this also applies to neurodegeneration. This is certainly one of the protective effects of terpenoids on nervous cells, acting as potent antioxidants and mitigating oxidative damage and inflammation (Table 4). The PI3K/Akt signaling pathway is an intracellular signal transduction pathway that promotes survival and growth in response to extracellular signals. For this reason, this pathway should be highly regulated by multiple mechanisms involving crosstalk with other signaling pathways. Therefore, it is important to note that several terpenoids are associated with PI3K/Akt signaling regulation, showing their high potential in the prevention of neurodegeneration (Table 4).

**Table 4:** Illustrative examples of terpenoids eliciting neuroprotective activities.

Terpenoid	Assay	Reported Activities	Ref.
Artesunate	C57BL/6 mice (newborn) and primary neural stem/progenitor cells (NSPCs)	Ameliorated the insufficient endogenous neural stem/progenitor cell (NSPC) proliferation caused by ischemia. By stimulating the PI3K/Akt signaling pathway, ART could increase the phosphorylation level of FOXO-3a, downregulate p27kip1, and inhibit the transcription of FOXO-3a.	[124]
Asiatic acid	Neuronally differentiated PC12 cells	Protection against Aβ25-35-induced apoptosis and tau hyperphosphorylation by regulating PI3K/Akt/GSK-3β signaling.	[125]
Asiaticoside	Streptozotocin (STZ)-induced diabetic cognitive deficit rat model	Ameliorated cerebral oxidative stress, inflammation, and apoptosis.	[126]
Catalpol	Streptozotocin-induced hyperglycemic mice	Antioxidant and neuroprotective effects on mouse models of depression, improving their depressive behavior by upregulating the PI3K/Akt/Nrf2/HO-1 signaling pathway.	[127]
	Stroke model and the primary neurons from the rat stroke model	Catalpol activated the PI3K/Akt/mTOR pathway, decreasing the expression of miR-124 and increasing the expression of downstream protein S6, thus enhancing in vivo axon growth and neuronal survival in stroke models.	[128]
Celastrol	Acute spinal cord injury rats	Inhibited microglial pyroptosis and attenuated inflammatory reactions.	[129]
Geniposide	Epileptic rats model	Activated Akt, followed by increased PI3K and GSK-3β expression, thus improving pathological symptoms.	[130]
	Hippocampal neurons	Inhibited apoptosis, resulting in antidepressant properties in the brain.	[131]
	Chronic constriction injury model of neuropathic pain	Inhibited the EGFR/PI3K/Akt signaling pathway, thereby alleviating pain symptoms in the sciatic nerve.	[132]
Bilobalide and ginkgolides	Rat model of middle cerebral artery occlusion	Supported neuronal cell survival in patients suffering from ischemic stroke.	[133]
	PC12 neuronal cells	Bilobalide derivatives (diAc-iso and diBrBn-iso) performed better than the original compound (proliferating cell activity, neuroprotective effects against Aβ (1–40) peptides, and neurite outgrowth effects).	[134]
Ginkgolide B	Rats cerebral I/R damage model	Activation of Nrf2 and CREB through PI3K/Akt signaling.	[135]
	Rat model of middle cerebral artery occlusion (MCAO) and OGD/R cell model	Antioxidant effects against cerebral ischemia injury by activating the Akt/Nrf2 pathway.	[136]
Ginkgolide K	Primary cortical astrocytes from newborn mice exposed to oxygen–glucose deprivation	Superior therapeutic potential to ginkgolide B; easier to upregulate PI3K and p-Akt expression, affecting downstream pathways, thereby contributing to anti-inflammatory and antioxidant effects.	[137]
Echinocystic acid	Collagenase-induced intracerebral haemorrhage mice	Neuroprotective effect via the PI3K/Akt pathway.	[138]
Methyl lucidone	HT-22 cell line	Neuroprotective effects on glutamate-induced oxidative stress in HT-22 cells via Nrf-2/HO-1 signaling.	[139]
Limonene	Maternal separation mice	Antidepressant-like effects due to the reduction of nitrite levels in the hippocampus.	[140]
Lycopene	Primary mouse neurons	Protected against T-BHP-induced neuronal oxidative damage and apoptosis via activation of the PI3K/Akt pathway.	[141]
Platycodin D	Primary cortical neurons	Protected cortical neurons against oxygen–glucose deprivation/reperfusion in neonatal hypoxic–ischemic encephalopathy.	[142]
Polygalasaponin F	-	Glutamate-induced cytotoxicity cell model/protects hippocampal neurons against glutamate-induced cytotoxicity.	[143]
	Rat adrenal pheochromocytoma cells (PC12) and primary rat cortical neurons	Inhibited neuronal apoptosis induced by oxygen–glucose deprivation and reoxygenation through the PI3K/Akt pathway.	[144]
Ginsenosides	Oxygen–glucose deprived (OGD) SH-SY5Y cells	Neuroprotective effect of panax notoginseng saponins by activating the EGFR/PI3K/Akt pathway.	[145]
	Mouse model	Protective effects of notoginsenoside R1 via regulation of the PI3K-Akt-mTOR/JNK pathway in neonatal cerebral hypoxic–ischemic brain injury.	[146]
α-Pinene	Focal cerebral I/R in rats	Neuroprotective effect during ischemic stroke by attenuating neuroinflammation/ELISA.	[147]

Although several studies have investigated the bioavailability and blood–brain barrier (BBB) permeability of terpenoid compounds, there are still many challenges inherent in using terpenoids to prevent/treat neurodegenerative diseases. Despite evidence of the neuroprotective potential of terpenoids, the scarcity of clinical evidence and research studies on the application of terpenoids as a treatment for neurodegenerative diseases poses a significant challenge. Mehri et al. [148] evaluated the linalool potential neuroprotective effect in ACR-induced neurotoxicity in Wistar rats using advanced analytical techniques. They concluded that linalool exhibited protective effects against ACR-induced neurotoxicity in Wistar rats through the elevation of GSH contents and the reduction of lipid peroxidation in the cerebral cortex [148]. Weston-Green et al. [149] examined the effects of two key terpenes, pinene and linalool, on parameters relevant to neurological and psychiatric disorders, reporting that linalool and pinene influence multiple neurotransmitter, inflammatory, and neurotrophic signals, as well as behavior, demonstrating psychoactivity (albeit non-intoxicating) [149] In addition, Sánchez-Martínez et al. [150] reported the in vitro neuroprotective potential of terpenes from industrial orange juice by-products through multiple in vitro assays, including a parallel artificial membrane permeability assay for the blood–brain barrier (PAMPA-BBB) [150]. Further investigation is necessary to determine the efficacy and safety of terpenoids for treating these conditions [151]. For instance, it is necessary to overcome the low bioavailability and ability to cross the BBB, which limits the effectiveness of terpenoids in the treatment of neurodegenerative diseases [152,153], as well as the lack of studies standardizing dosage and formulation, making it difficult to determine the appropriate dose for patients [154]. Moreover, the mechanisms of action of terpenoids are complex and not fully understood, jeopardizing target identification for treatment [152] and the identification of potential drug interactions and side effects [155].

## 4. Industrial Applications of Terpenoids

**Figure 5:**
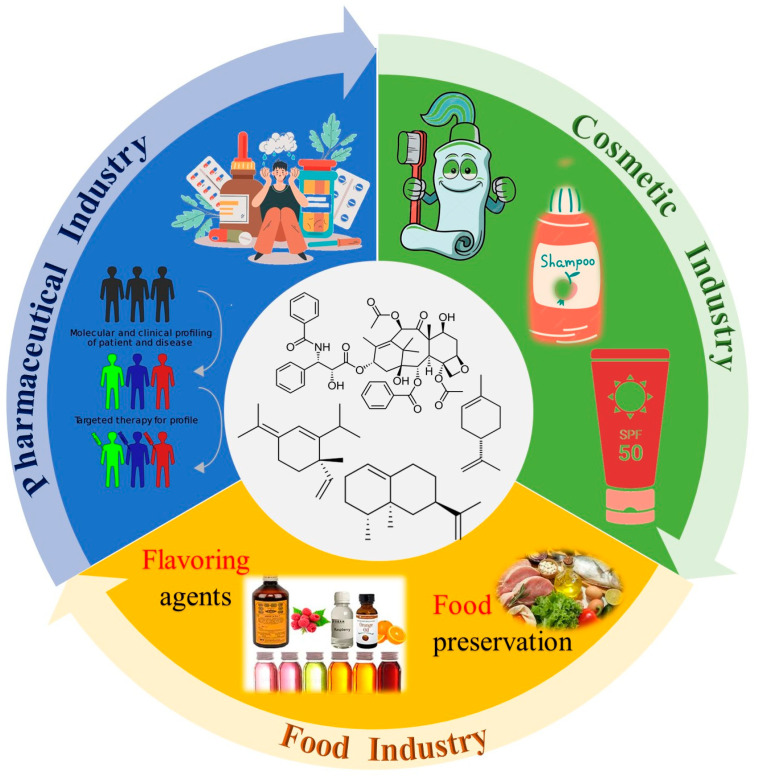
Potential industrial applications of terpenoids.

Terpenoids have been widely investigated for their industrial applications, including in pharmaceutical, cosmetic, food, and other industries. They are used as new anticancer drugs, natural flavoring compounds, natural food preservatives, pesticides/insecticides in the agricultural industry, and raw materials in the production of industrial chemicals (e.g., surface preservatives), as well as in the aerospace and automotive industries, among others. This diversity of terpenoids’ industrial applications results from their impressive antiviral, antimalarial, anti-inflammatory, and antibacterial properties, making them valuable ingredients for industrial applications. In the following subsections, we will present some findings related to the application of terpenoids in the pharmaceutical, cosmetic, medical, and food industries (Figure 5) [46].

### 4.1. Pharmaceutical and Medical Industry

Terpenoids are widely used in clinical medicine as new anticancer drugs in the pharmaceutical industry since they possess antitumor, anti-inflammatory, and anticancer properties. Given these properties, terpenoids have also shown the potential to be used as new therapeutic agents to treat or prevent malaria, inflammation, pain, cardiovascular diseases, and other diseases [46].

Global Cancer Data 2020 estimates that there were 19.3 million new instances of cancer and 10.0 million cancer deaths globally this year, making the current cancer rates a serious global public health issue [156]. The pharmaceutical industry has placed a significant emphasis on research into active anticancer and cancer prevention components in its quest to find treatments for malignant tumors. They provide new active molecules for the development of drugs, such as taxol, elemene, selenodiene, and limonene, which exert their antitumor and anticancer activities through different or novel mechanisms [157]. Taxol, a natural diterpenoid, was discovered and refined from Taxus plants and appears as a novel class of antitumor medications and is used in the treatment of advanced types of cancer (e.g., ovarian, breast, and lung cancer) [158]. Elemene, a newly developed and widely used anticancer medication produced from Curcuma wenyujin, has very mild side effects in the treatment of lung cancer, liver cancer, nasopharyngeal carcinoma, and brain tumors and does not include harmful epoxy, nitro, onion ring, or benzene ring groups [159]. This is a sesquiterpene compound with low toxicity, a broad spectrum, and fewer negative effects than radiation and chemotherapy, while improving the effectiveness of treating solid cancers. Other studies have shown that elemene has a sizable inhibitory impact on cancer cells and that it inhibits lipid-induced inflammatory pathways in heart failure [160]. Regarding limonene, there are two optically active forms: l-limonene, and d-limonene. d-limonene is the most common form and has the strongest pharmacological action. It is used in the field of medicine for the treatment of cholecystitis, a condition characterized by inflammation of the gallbladder [161]. Moreover, the anti-inflammatory and antitumor properties of limonene are primarily caused by the inhibition of pro-inflammatory molecules that are linked to cancer [162]. Pinene has anti-inflammatory and antiapoptotic properties and has been found to be effective in the treatment of Alzheimer’s disease, Parkinson’s disease, and other neurodegenerative diseases [163]. On the other hand, artemisinin, a sesquiterpene lactone, has been found to have antimalarial functions; consequently, artemisinin derivatives have been used as a therapeutic agent for malaria since they contain endoperoxide bridges, which are essential for antimalarial activity [164]. This sesquiterpene lactone destroys malaria parasites by inhibiting the proteasome and damaging proteins [165].

However, the application of terpenoids in the pharmaceutical and medicinal industries faces challenges such as complex sources, low yield, and low purity. The production of terpenoids by microorganisms has drawn more interest recently because of the limits of natural sources. Large-scale fermentation of terpenoids using engineered microbes promises a better output, batch-to-batch uniformity, reduced cost, and more sustainability. Moreover, the safety and pharmacological activity of terpenoids in medicine have always been a focus and a difficult problem to solve. Further exploration of the mechanisms of action of terpenoids in drugs should provide a better grasp of their pharmacological effects. The clinically evident method of therapeutic activity of many terpenoids has not yet been clarified, and further research is needed to develop new medication substances [166].

### 4.2. Cosmetics Industry

Terpenoids have been used in the cosmetics industry as major aroma components and for their various beneficial properties. They contribute substantially to the overall market value of the global cosmetic products market, which was valued at over USD 500 billion in 2017, since they are incorporated into products such as hair tonics, soap, and toothpaste, among others [167]. Due to their scent and advantages in enhancing attractiveness by making the skin wrinkle-free, healthy, and radiant, tocols, carotenoids, and some terpenoids (e.g., limonene, linalool, and eucalyptol) are extremely popular as ingredients in the cosmetics industry [167]. They are frequently utilized in cosmetics due to their antioxidant properties in reducing melanogenesis, UV radiation damage to skin cells, and aging. Considering the benefits they provide and their practical use in the cosmetic industry, a study has concentrated on the role of different carotenoids, including β-carotene, astaxanthin, lycopene, and fucoxanthin [168]. Recent research has shown that lycopene from tomatoes, along with phytoene, phytofluene, tocopherols, and phytosterols, can act as a photoprotectant by preventing the UVR from increasing the expression of the genes for matrix metallopeptidase 1, intercellular adhesion molecule 1, and heme oxygenase 1 genes [169]. Moreover, limonene has been extensively used in the cosmetics industry and skin care as a fragrant ingredient due to its pleasant citrus fragrance. Nevertheless, this monoterpenoid can cause skin dryness and irritation, and oxidized limonene can cause allergic contact dermatitis and counts as a frequent skin sensitizer [170]. 

### 4.3. Food Industry

Terpenoids are widely used in the food industry as natural preservatives, flavoring agents, sweetening agents, and functional food ingredients [171]. Regarding natural food preservatives, terpenoids are significant phytochemicals with a wide spectrum of antioxidant and antimicrobial activities that are found in a wide variety of fruits and vegetables. The bioactivity of these phytochemicals as antioxidants allows them to scavenge reactive oxygen and nitrogen species (e.g., superoxide, hydroxyl, and peroxyl radicals) from meat and other products, which helps to avoid the oxidation of lipids in food products (e.g., meat, fish, fruits, and vegetables). This makes them extremely helpful in food preservation [172]. In this sense, monoterpenoids from EOs from *Lamiaceae* plants, which demonstrate a high antioxidant activity, have been investigated in recent years as natural alternatives to prevent the oxidation of food-related products [173]. Research conducted by Rodriguez-Garcia et al. [174] investigated the influence of oregano EO (composed of 47.4% carvacrol, 26.4% *p*-cymene, and 3.02% thymol) applied within pectin coatings on the inhibition of *Alternaria alternata* growth, sensorial acceptability, and antioxidant content of tomatoes. The findings demonstrated that the antifungal and antioxidant activities of pectin–oregano EO coatings were enhanced without having a detrimental impact on the sensory acceptability of tomatoes.

Terpenoids like linalool, α-pinene, limonene, and geraniol have been designated as “generally recognized as safe” (GRAS) by the United States Food and Drug Administration (FDA) and are widely used as natural flavoring agents in the food industry. Geraniol, one of the most in-demand monoterpenoids, with an amusing rose-like odor, has been used as a flavoring additive in chewing gum, candy, ice cream, beverages, and many other products [171]. Nerolidol is a classic sesquiterpene, formally allowed as a food flavoring additive by the US FDA, giving a wood- and fresh-bark-like scent [175]. With a market volume of roughly 10,000 kg annually, valencene is another sesquiterpenoid utilized commercially as an addition to give or reinforce a fruity and woody flavor in foods and beverages. Additionally, a few different terpenes with distinct flavors, discovered in a variety of plants, have been explored not only by the food industry but also for their role in consumer products like pesticides and insecticides, among others.

## 5. Green Emerging Extraction Techniques

Sample preparation is a key step in studies involving the discovery of active compounds. The extraction procedure should be adapted to the nature of the target compounds through the use of rapid, simple, and automatic methods for higher reproducibility [176]. After extraction, purification methods are used to isolate the compounds of interest, which depend on the terpenoid structure, chemical characteristics, physical quality, and amount of initial plant material [48,177]. Therefore, the properties of a particular terpenoid define the optimum extraction procedure [48,177].

Conventional extraction methods include maceration, Soxhlet extraction, solvent extraction, and hydrodistillation [176,178]. However, these techniques often require a large volume of organic solvents, which harm the environment, are time-consuming, can lead to the thermal degradation of some natural products, and have a low extraction efficiency [176]. Due to growing environmental concerns, green extraction techniques that aim for maximal extraction at a low cost while using environmentally friendly methods have received much interest in recent years [179].

Innovative techniques such as pressurized liquid extraction (PLE), static headspace (HS) extraction, microwave-assisted hydrodistillation (MAHD), and supercritical fluid extraction (SFE) have been developed (Table 5) [46]. These techniques use smaller volumes of solvent and have lower extraction times, better selectivity, and higher efficiency compared to conventional methods [176]. In general, these techniques comprise (i) breakage of plant cells to release their chemical constituents; (ii) sample extraction using a suitable solvent or through distillation or compound trapping; (iii) separation of the target compound from undesired contents of extracts; and (iv) analyses of the product using an appropriate method. Furthermore, the optimal extraction method depends on the properties of the targeted terpene [46]. The final step consists of the identification, characterization, and authentication of the extracted compound. Methods such as thin-layer CT (chromatostrip), high-performance counter-current chromatography (HPCCC), liquid chromatography (LC), HPLC with an ultraviolet detector (HPLC-UV), gas chromatography–mass spectrometry (GC-MS), and ultra-HPLC (UHPLC) are often used [46,180].

### 5.1. Pressurized Liquid Extraction (PLE)

PLE operates at high pressures (35–210 bar) and temperatures above the boiling points of solvents (50–200 °C). The use of higher temperatures decreases the solvent’s viscosity and allows for a better solubility of the compounds in the solvent. The use of high pressures allows for better penetration of the solvent in the matrix and thus a better extraction of the analytes [176]. Moreover, pressurized solvents have increased diffusivity, solubility, viscosity, and dielectric constant and remain in a liquid state, which facilitates their extraction at a higher temperature. These properties can be modified by changing the temperature and pressure [181]. PLE consumes small solvent amounts, significantly decreases the extraction time, and can be automated [176]. However, this technique requires high extraction temperatures and large and sophisticated equipment [181]. Sánchez-Martínez et al. [182] extracted terpenoids from *Citrus sinensis*. The authors also analyzed the neuroprotective potential of the PLE extracts through in vitro assays. The GC quadrupole time-of-flight MS (GC-q-TOF-MS) analysis revealed that the mono- and sesquiterpenoids showed the highest neuroprotective capacity. Furthermore, the selected extracts showed high antioxidant, anticholinesterase, and anti-inflammatory properties, with low cytotoxicity and protection against L-glutamic acid in cell models (Table 5).

Wang et al. [183] studied the impact of PLE and dimethyl sulfoxide (DMSO) concentration (0%, 30%, 50%, and 100%) on the antioxidants and minerals yield from *Chlorella*. They found that 100% DMSO allowed for the extraction of antioxidants and pigments from *Chlorella* (polyphenols 10.465 mg/g, chlorophyll a 6.206 mg/g, chlorophyll b 3.003 mg/g, and carotenoids 0.971 mg/g) and was thus the chosen concentration for the recovery studies on *Spirulina*, *Chlorella*, and *Phaeodactylum tricornutum*. Fucoxanthin, all-*trans*-β-carotene, diatoxanthin, lutein, and (9*Z*)-β-carotene were the main carotenoids in *P. tricornutum*. In *S. maxima*, β-carotene, zeaxanthin, (9*Z*)-β-carotene, and myxoxanthophyll were the most predominant; while in *C. vulgaris*, the most predominant compounds were lutein, α-carotene, β-carotene, and (9*Z*)-β-carotene.

**Table 5:** Green innovative techniques employed for the extraction of terpenoids found in the literature over the last 5 years.

Matrix	Extraction Conditions	Main Conclusions	Ref.
**Pressurized liquid extraction (PLE)**			
Orange juice by-products	Amount of 4 g orange powder residue + 8 g sea sand (1:2 *w*/*w*) placed into the extraction cell; 25 mL ethyl acetate; 96 °C, 30 min, 10 MPa, on static mode; 1 min of N_2_ purging; extracts stored in −20 °C in dark before drying; GC-q-TOF-MS analysis.	Terpenoids revealed promising neuroprotective action. Antioxidant activity: ABTSIC50 = 13.5 μg/mL; ROSIC50 = 4.4 μg/mL. Anticholinesterase activity: AChEIC50 = 137.1 vg /L; BChEIC50 = 147.0 μg/mL. Anti-inflammatory properties: against IL-6 and LOXIC50 = 76.1 μg/mL, with low cytotoxicity and protection against L-glutamic acid in cell models.	[182]
Microalgae *Spirulina*, *Chlorella,* and *Phaeodactylum tricornutum*	Microalgae and diatomaceous earth completely mixed (0.5 g: 1.5 g) in a mortar and placed into PLE extraction tank ASE-200 Accelerated Solvent Extractor (preheating for 1 min, heating time of 5 min, flush volume 60%, N_2_ for 60 s, 103.4 bars, 40 °C, 15 min); 100% DMSO; HPLC analysis.	The authors found that 100% DMSO allowed for the extraction of antioxidants and pigments from *Chlorella* (polyphenols 10.465 mg/g, chlorophyll *a* 6.206 mg/g, chlorophyll *b* 3.003 mg/g, carotenoids 0.971 mg/g) and was thus the chosen concentration for the recovery studies on *Spirulina*, *Chlorella,* and *Phaeodactylum tricornutum*. Fucoxanthin, β-carotene, zeaxanthin, and lutein were the main carotenoids found in *P. tricornutum*, *Spirulina,* and *Chlorella*, respectively.	[183]
**Static headspace (HS) extraction**			
Cinnamon, thyme, cumin, fennel, clove, nutmeg, and orange	Amount of 20 mg of spice or 2 g of orange peels placed in a 20 mL headspace vial; 125 °C, 30 min, 250 rpm; Combi-pal + automatic HS injector; GC-MS analysis (1 mL); HS syringe heated at 130 °C.	Static HS extraction allowed for the recovery of extracts with higher concentrations in comparison with hydrodistillation and PLE. For example, eugenol LOD: Static HS: 0.0022 µg/g; PLE: 0.03 µg/g.	[176]
Cannabis	A total of 5 mg powder samples placed in a 20 mL amber rounded bottom HS vial; CTC autosampler used with an HS static tool in splitless mode; 40 min, 140 °C, 250 rpm; GC-MS/MS analysis (600 μL).	Ninety-three terpenoids were identified. Sample preparation methods significantly impacted the chemical fingerprint of the samples when compared to non-treated *Cannabis.* Static HS extraction allowed for the quantification of natural terpenoid contents of chemovars.	[184]
Citrus leaves	A total of 1 g powder sample placed in a 20 mL HS vial + 30 μL internal standard (0.1% *n*-hexanol); sealed vials mixed thoroughly before being placed on a static 7697A HS auto-sampler, awaiting injection; 15 min incubation at 100 °C; GC-MS analysis.	A total of 83 volatile metabolites were identified, including monoterpene hydrocarbons (68.23–95.08%, 21 compounds), alcohols (0.69–26.0%, 8 compounds), sesquiterpene hydrocarbons (0.47–5.04%, 26 compounds), aldehydes (0.12–11.26%, 10 compounds), monoterpenoids (0–0.36%, 7 compounds), esters (0–0.18%, 5 compounds), ketones (0–0.02%, 2 compounds), and miscellaneous compounds (0–1.11%, 4 compounds).	[185]
**Microwave-assisted hydrodistillation (MAHD)**			
Hop (*Humulus lupulus* L.)	MAHD was carried out using ETHOS X and ETHOS XL extractors; GC-MS analysis.	The highest extraction yield was obtained for fresh hops (20.5 mL_VF_/kg_dry matrix_). When 3 kg of the sample were used, this value achieved a value of 17.3 mL_VF_/kg_dry matrix_. In a pilot reactor (30 kg capacity), high yield increases were seen for pelletized and dried samples in quadruple and double the lab-scale yields, respectively.	[186]
Sage herbal dust	Amount of 40 g dry plant material + 400 mL distilled water; MAHD performed in the oven (90, 180, 360, 600, and 800 W) for 2 h; water–oil mixture evaporated through glass pipe connector to be condensed in Unger apparatus; essential oils collected and stored at 4 °C until analysis; GC-MS analysis.	A total of 55 terpenoids were identified. The main compounds in the essential oils are obtained via the following methods: Hydrodistillation—viridiflorol (21%), camphor (16.54–19.05%), and α-thujone (11%); MAHD at 90W—camphor (24.88%), α-thujone (22.21%), and eucalyptol (18.37%); MAHD at 360W—viridiflorol (33.27%) and verticiol (13.71%) (in other MAHD samples, viridiflorol (17.17–23.7%) and camphor (14.46–18.82%)).	[187]
Withered flowers of *Magnolia soulangeana* Soul.-Bod.	MAHD with uniform heating (623 W, 54 min, 60 r/min); 50 g soaked raw materials + distilled water in a 500 mL distillation flask (6.4 mL/g liquid–solid ratio); withered flowers soaked for 8 h before essential oil preparation; anhydrous sodium sulfate added to remove the moisture; sample transferred to a low-temperature environment (4 °C ± 2 °C) for storage; GC-MS analysis.	The introduction of the rotation unit and soaking pretreatment unit increased the yield of essential oil by 16.67% and 20%, respectively. This method showed a lower energy consumption and environmental pressure than conventional approaches for essential oil preparation. The samples obtained were rich in terpenes (49.32%), such as eucalyptol, δ-cadinene, α-muurolene, and germacrene D. δ-cadinene was the main compound to exert hypolipidemic activity.	[188]
Lavenders (*Lavandula x intermedia var. Super A*)	Dried lavenders grinded at 6000 rpm for 10 s and subjected to soaking (1:10, w/v) for 1 h before extraction process. Essential oils of dried lavender extracted via MAHD (ETHOS X) at 750 W for 2 h; GC-MS analysis.	Lavender essential oil yield was around 5.5%. Based on the GC-MS data, major constituents of linalool L (29.0%), 1,8-cineole (13.9%), camphor (12.3%), and linalyl acetate (11.9%) were the main compounds identified.	[189]
Peppermint	Amount of 40 g dry plant material + 400 mL distilled water; MAHD performed in the oven (180, 360, 600, and 800 W) for 2 h; water–oil mixture evaporated through glass pipe connector to be condensed in Unger apparatus; essential oils collected and stored at −18 °C until analysis; GC-MS analysis.	Monoterpenes were the main class of compounds in all samples with menthol (33.07–37.43%), menthone (9.49–25.21%), isomenthol (4.27–10.21%), isomenthone (4.51–6.06%), and eucalyptol (1.16–4.89%). Sesquiterpenes were also predominant with *trans*-caryophyllene (4.58–10.56%) and germacrene D (2.65–7.65%).	[190]
**Supercritical fluid extraction (SFE)**			
Flesh and peels of 15 matrices of vegetables	Amount of 5 g freeze-dried samples + 95 g inert glass beads; 15 g/min CO_2_; 30 min, 59 °C, 350 bar, 15.5% (*v*/*v*) ethanol as co-solvent; extracts collected and dissolved in ethanol and stored at −18 °C in dark glass containers until analysis; HPLC analysis.	TCR values higher than 90% *w*/*w* for most samples. β-carotene was the most successfully extracted compound (TCRs 88–100% *w*/*w*). More polar carotenoids, such as lutein and lycopene, exhibited lower TCRs.	[191]
Mango peel	Amount of 5 g mango peel + 6.7 g/min CO_2_; 180 min, 60 °C, 25 MPa, 15.5% (*w*/*w*) ethanol as co-solvent; after extraction, remanent ethanol evaporated under vacuum (35 °C, 100 mBar); dried extracts stored at −20 °C until analysis; RP-UHPLC-DAD analysis.	The extracts provided better protection to sunflower oil against lipid oxidation than all-*trans*-β-carotene when evaluated between 200–1000 ppm, which contained 6–28 ppm of all-*trans*-β-carotene.	[192]
Annatto seeds	Two-step sequential SFE extraction:1st step: 60 °C, 10 MPa, 290 kg/m^3^ CO_2_ to recover the geranylgeraniol-rich fraction; 2nd step: 40 °C, 20 MPa, 840 kg/m^3^ CO_2_ to recover the tocotrienols-rich fraction. Amount of 50 g annatto seeds packed in the extraction vessel + empty space filled with glass beads; 9.5 g/min CO_2_.	Different operational extraction conditions (temperature and pressure) resulted in extracts with different chemical compositions. The extract obtained at low CO_2_ density (290 kg/m^3^) produced a fraction enriched in geranylgeraniol with a low tocotrienols content. A two-step sequential SFE extraction process was employed to obtain a geranylgeraniol-rich fraction followed by a tocotrienols-rich fraction.	[193]
Carrot peels and flesh	Amont of 5 g dried peels + 95 g inert glass beads; 80 min, 59 °C, 349 bar; 15 g/min CO_2_; ethanol as co-solvent (15.5%); HPLC analysis.	β-Carotene represented 60% of the TCC in both flesh and peel, followed by α-carotene (30% of the TCC in both samples). In the peels, these two carotenoids accounted for almost 95% of TCC. Lycopene and lutein were also identified (1.9–30.2 μg/g). The optimum extraction conditions allowed for a carotenoid recovery of 86.1%. At 58.5 °C, 306 bar, and 14.3% ethanol, the processes retrieved maximum mass yield (5.31%, d.b.).	[192]
Leaves of *Piper klotzschianum*	Aount of 20 g leaves + the remaining extraction cell space filled with inert glass beads; after reaching 79.85 °C, the pump and extractor were simultaneously pressurized (220 bar); system left at rest to reach equilibrium (30 min); extraction was then performed up to 280 min; GC-MS analysis.	At optimum conditions, the highest extraction yield was 1.36%. The addition of organic co-solvents (5% of methanol) significantly improved the extraction yield to 2.18%, representing an increase of 40% compared to extraction using CO_2_ alone.	[194]
Caraway seeds	SWE: 1 g caraway + 2 g diatomaceous earth + 2 cellulose filter papers; sample cell placed in the oven; pump delivered solvent to the sample; cell heated to the set temperature under high pressure, and the extraction was performed for the designated time; after extraction, solvent purged out of the cell using N_2_ gas; extract collection. LLE: water extract + 20 mL *n*-hexane. Centrifugation (5 min); obtained extract stirred briefly and centrifuged (5 min); *n*-hexane transferred to an empty conical tube and stored in the freezer; GC and GC-MS analysis.	In SWE, smaller amounts of terpenes (limonene, carveol, and carvone) were found. The limonene concentration was higher for hydrodistillation (5 mg/g_caraway_) than for SWE. The carvone yield was higher when using SWE (28.5 mg/g_caraway_) than for solvent extraction (20.2 mg/g_caraway_) and hydrodistillation (19.8 mg/g_caraway_).	[195]

Legend: DMSO—dimethyl sulfoxide; LLE—liquid–liquid extraction; N_2_—nitrogen; SWE—subcritical water extraction; RP—reversed phase; TCR—total carotenoid recovery; TCC—total carotenoid content; VF—volatile fraction.

### 5.2. Static Headspace (HS) Extraction

The static HS technique consists of the extraction of volatile and semi-volatile compounds directly from the gaseous phase above the solid or liquid matrix. HS is a simple, rapid, solvent-free technique, with no sample preparation needed; it is automated and easily coupled to GC. This method allows for the analysis of small amounts of volatile compounds [176]. Recent reports suggested that HS is the most efficient extraction method used to recover terpenoids from various food matrices, compared to hydrodistillation and PLE, since this method allows for the direct measurement of plant materials without any kind of sample preparation other than grinding, which may lead to alterations in the content of terpenoids. Additionally, when compared to liquid injections, static HS reduces the contamination of the MS detector, liners, and columns [184]. Triaux et al. [176] used hydrodistillation as the reference method to compare the efficiency of extraction of the PLE and HS methods for the recovery of terpenes and terpenoids from various food matrices (cinnamon, thyme, cumin, fennel, clove, nutmeg, and orange). HS was the most efficient extraction method, yielding the most concentrated extracts. For instance, the eugenol LOD via HS was 0.0022 µg/g compared to 0.03 µg/g via liquid injection. Shapira et al. [184] quantified 93 terpenoids in cannabis air-dried inflorescences and extracts. The method consisted of the full evaporation of samples via a static HS sampler followed by GC-MS/MS analysis. The authors also compared the developed method with decarboxylation, evaporation, and extraction methods. The results showed that sample preparation methods significantly impacted the chemical fingerprint of the samples when compared to non-treated cannabis, which emphasizes the importance of performing static HS extraction to study the natural terpenoid contents of chemovars. Deng et al. [185] established the volatile profile of citrus leaves from 42 different cultivars. This study also evaluated the influence of four HS heating temperatures (40, 60, 80, and 100 °C) and two HS equilibration times (15 and 30 min). It was found that the extraction efficiency increased with rising temperature due to the volatilization of compounds from the matrix to the overlying HS. Moreover, at low heating temperatures, such as 40 and 60 °C, prolonging the incubation time from 15 to 30 min increased the amounts of extracted compounds. Based on these observations, the authors established that the optimal HS extraction conditions were a 15 min incubation at 100 °C. A total of 83 volatile metabolites were identified, namely, monoterpene hydrocarbons (68.23–95.08%, 21 compounds), alcohols (0.69–26.0%, 8 compounds), sesquiterpene hydrocarbons (0.47–5.04%, 26 compounds), aldehydes (0.12–11.26%, 10 compounds), monoterpenoids (0–0.36%, 7 compounds), esters 0–0.18%, 5 compounds), ketones (0–0.02%, 2 compounds), and miscellaneous compounds (0–1.11%, 4 compounds).

### 5.3. Microwave-Assisted Hydrodistillation (MAHD)

MAHD consists of the absorption of microwave electromagnetic energy by polar molecules inside the plant material through dipole rotation and ionic conductance. These processes increase the pressure inside the cells, leading to the disruption of membranes and the release of volatile bioactive compounds, which are subsequently recovered through a condensation system. MAHD ensures more rapid heating using the water inside the plant matrix without the need for additional water, allowing for energy and water savings [196]. Hence, MAHD has been developed to reduce energy and time in lab-scale reactors up to industrial-scale systems [186]. This technique shows reduced extraction times, requires less solvent, and allows for a higher recovery of EOs compared to traditional methods [46,196]. Moreover, the biomass is treated more homogeneously than in classical distillation since the irradiation is not focused on the bottom of the reactor, as occurs in classical distillation [186]. Lamberti et al. [186] investigated the volatile fraction (VF) of fresh, dried, and pelletized hops using MAHD followed by GC-MS for their characterization. The optimized lab-scale protocol yielded the best extraction value of 20.5 mLVF/kg of dry matrix for fresh hops. However, when the highest matrix amount was used (3 kg), the extraction yield was reduced to 17.3 mLVF/kg of dry matrix. Then, the authors test a pilot reactor able to process 30 kg of sample. In this case, the yields increased to quadruple and double the lab-scale yields in pelletized and dried products, respectively. Radivojac et al. [187] recovered terpenoids from sage herbal dust through MAHD and compared them with conventional hydrodistillation. A total of 55 terpenoids were identified in the EOs of sage herbal dust via GC-MS analysis. The authors found that the chemical composition of the EOs differed significantly according to the method used. This could be explained by chemical alterations that occur during distillation, such as oxidation and hydrolysis. Despite this, the main compounds were present in all samples. Viridiflorol (21%), camphor (16.54–19.05%), and α-thujone (11%) were the main compounds obtained via hydrodistillation. Moreover, ledene oxide-(II), caryophyllenyl alcohol, o-cymen-5-ol, widdrol, and longifolenaldehyde were only identified in the sample subjected to this method, where they represented 6% of the sample. In MAHD, at 90W, camphor (24.88%), α-thujone (22.21%), and eucalyptol (18.37%) were the main compounds; while at 360W, the main compounds were viridiflorol (33.27%) and verticiol (13.71%). In other MAHD samples, viridiflorol (17.17–23.7%) and camphor (14.46–18.82%) were the most predominant compounds. Aromadendrene oxide, phenylethyl alcohol, globulol, alloaromadendrene, and linalyl acetate made up 9.64% of the MAHD samples. Pavlic et al. [190] applied both traditional and emerging extraction techniques to obtain peppermint extracts and EOs. Conventional hydrodistillation and MAHD were used for the recovery of EOs, which retrieved a high content in terpenoids, namely, menthol, menthone, isomenthol, eucalyptol, and isomenthone (Table 5). Due to the high concentration of terpenoids in these samples, the EOs were revealed to reduce the power of cupric ions, the chelating of metals, and total antioxidant activity. Soxhlet extraction, SFE, microwave-assisted hydrodistillation, and UAE were applied for the non-selective recovery of peppermint lipophilic extracts. SFE extracts were useful for the green production of solvent-free peppermint extracts rich in terpenoids and other lipophilic bioactives. MAHD was identified as an alternative to traditional methods of recovering EOs.

### 5.4. Supercritical Fluid Extraction (SFE)

SFE consists of the use of a dense gas or compressed liquid lacking any molecular interactions. The supercritical fluid exhibits properties of both gas (mass transfer, diffusion, lower viscosity, high penetration power) and liquid (high density, solvent power, reduced surface tension, and viscosity) at temperatures and pressures above critical points, with tunable density [181,197]. SFE is a fast, efficient, economical, selective, and simple procedure for the extraction of terpenoids [198]. Carbon dioxide (CO_2_) and water are the two commonly used supercritical fluids in the industry on a large scale. However, the corrosive nature of water limits its use in the food and pharmaceutical industries [181,198]. SFE with CO_2_ as the solvent uses an essentially non-toxic solvent with low potential for the formation of interferents. Moreover, CO_2_ has a low critical temperature and a high density, which makes it suitable for the extraction of heat-labile compounds and a high-dissolving power, respectively [181,198]. Supercritical CO_2_ extraction (SCO_2_) has a low polarity index, making it useful for dissolving lipophilic compounds such as Eos but unsuitable for polar compounds [181]. Therefore, the inclusion of polarity modifiers such as ethanol (EtOH), as well as the development of SFE apparatus capable of producing pressures over 600 bar, enables the extraction of several intermediate polarity compounds [45]. Lima et al. [194] extracted valuable chemical compounds from dry leaves of *Piper klotzschianum* by SCO_2_. The sesquiterpenes were the most representative class in the extracts, in which the major constituents found were germacrene D, pipercallosidine, 14-oxy-α-muuroleno, bicyclogermacrene, and (*E*)-caryophyllene. The authors found that when using a co-solvent, the polarity increased, which resulted in an increased yield of some compounds due to a slight decrease in the percentage of hydrocarbons extracted. In another study, Andrade Lima et al. [191] evaluated carotenoid recovery from 15 carotenoid-rich fruit and vegetable wastes. SFE allowed for the extraction of carotenoids from a mixed sample of fruit and vegetable matrices and provided high recovery levels, which ranged from 74% to 99% *w*/*w*. The authors also found that the carotenoid extraction appeared to be influenced by the polysaccharide and moisture content of vegetable matrices. Kim et al. [195] developed a method for the extraction of oxygenated terpenes from caraway using subcritical water extraction (SWE). Each terpenoid’s chemical structure had a different effect in its extraction. For instance, the ideal conditions for carvone extraction were 170 °C and 15 min, while those for limonene were 110 °C and 10 min. SWE resulted in a higher carvone yield (28.5 mg/g caraway) than solvent extraction (20.2 mg/g caraway) and hydrodistillation (19.8 mg/g caraway). This study also showed that oxygenated terpenes such as carvone had a high solubility in subcritical water, while non-oxygenated terpene like limonene could partially be converted to carvone through SWE.

## 6. Concluding Remarks

Terpenoids are the largest group of secondary metabolites, highly diverse in properties and chemical structure. As semiochemicals, terpenoids play a significant role in mediating interactions between plants and insects, acting as a chemical defense mechanism against herbivorous insects via the synthesis and emission of volatile organic compounds that can directly affect herbivores’ physiology and behavior due to their toxic and repelling properties. In addition, plants produce terpenoids to attract specific insects that play a role in their pollination or seed dispersal.

Moreover, terpenoids have been largely associated with a wide range of biological activities, including antioxidant, antimicrobial, antifungal, anti-inflammatory, antiallergic, antiangiogenesis, and antimetastatic activities, among others. They have been shown to exert antitumorigenic effects, suggesting their potential use as chemotherapeutic agents for treating tumors. Terpenoids have been reported as effective in the prevention of cardiovascular diseases by alleviating cardiovascular-related complications, such as high blood pressure, atherosclerosis, and impaired heart function, by modulating inflammation and apoptosis via the MAPK pathway. The antifungal activity of terpenoids might be caused by their highly lipophilic nature and low molecular weight, causing cell death or inhibiting the sporulation and germination of food spoilage fungi. They have also shown antimicrobial activities against both antibiotic-susceptible and antibiotic-resistant bacteria and are involved in suppressing microglia-mediated inflammation in acute or chronic inflammatory diseases. Terpenoids have also been widely investigated for their industrial applications, including in the pharmaceutical, cosmetical, and food industries. They are used as new anticancer drugs, natural flavoring compounds, natural food preservatives, pesticides/insecticides in the agricultural industry, and raw materials in the production of industrial chemicals.

## Figures and Tables

**Table 2:** Representative examples of terpenoids with anticancer activities reported in the literature over the last five years.

Terpenoid (Source)	Cancer Type Assay/Reported Activities	Ref.
Andrographolide (*Andrographis paniculate*)	C8161 and A375 human malignant melanoma cells/cell cycle arrest and apoptosis	[53]
Betulinic acid	A375 melanoma cells/dose-dependent inhibitory effect in both mitochondrial respiration and glycolysis; induced mitochondrial dysfunction (10 μM)	[54]
Borneol	Glioma cells/promote apoptosis through the regulation of HIF-1a expression via theMTORC1/EIF4E pathway; esophageal squamous cell carcinoma cells/enhances paclitaxel-induced apoptosis via the inactivation of the PI3K/AKT pathway	[55,56]
Bornyl cis-4-hydroxycinnamate	Melanoma cell/apoptosis via mitochondrial dysfunction and endoplasmic reticulum stress	[57]
(+)-Bornyl *p*-coumarate	Melanoma cells/induced apoptosis and autophagy	[58]
Carvone	MCF-7 breast ductal carcinoma/Protective effect against tumor (IC_50_ 14.22 μM)	[59]
Citral (nanostructured lipid carrier)	MBA-231 breast cancer cells/in vitro cytotoxicity and anticancer activity	[60]
Chlorinated guaiane-type sesquiterpene lactones (*Centaurea* plants)	U-937 leukemia HL-60 cells/cytotoxicity	[61]
Curcumin and crocin	Cervical cancer cells/protective and therapeutic effects against tumor cells	[62]
Cucurbitacin C (Cucumber)	Cancer-cell-derived xenograft tumors in athymic nude mice/dose-dependent inhibited proliferation and clonogenic potential: cell cycle arrest at G1 or G2/M stage at low-dose; apoptosis at high-dose treatment	[63]
Cucurbitacin E	NCI-N87 gastric cancer cells/enhanced doxorubicin cytotoxicity	[64]
Helenalin	Embryonal rhabdomyosarcoma cells/increase ROS levels, decrease mitochondrial membrane potential, trigger endoplasmic reticulum stress, and deactivate the NF-κB pathway	[65]
Hinokitiol (>90%)	A549 lung adenocarcinoma/reduced cell migration and chemoprevention	[66]
Limonene (chitosan nanoparticles containing limonene and limonene-rich essential oils)	Melanoma and breast cancers/potential phytotherapy agents for cancer treatment	[67]
Myrcene	A549 lung adenocarcinoma/increased apoptosis via caspase induction (IC_50_ 0.5 μg/mL), MTT assay	[68]
Myrtenal	B16F0, B16F10, and SkMel-5 melanoma cells/decreased tumor cells migration and invasion	[69]
Oleanolic and ursolic acid derivatives	NCI-60 cancer cells/antiproliferative and cytotoxic effects	[70]
Thymoquinone	B16-F10 melanoma cells model/inhibition of melanogenesis	[71]
Triptolide	Breast cancer cells/enhanced sensitization to doxorubicin (DNA damage response inhibition)	[72]
α-Phellandrene	Melanoma (B-16/F-10), and murine (S-180) cells/antinociceptive and tumor-reducing effect (CI50 436.0 and 217.9 μg/mL); MTT assay	[73]
α-Pinene	HepG2 liver cancer/reduced cell growth (IR 39.3%), MTT assay	[74]
α-Terpineol	Murine sarcoma 180 cell line/antitumor activity against different tumor cell lines (lung, breast, leukemias, and colorectal); blockage of NF-kB expression	[75]
α-Thujone (*Thuja occidentalis* L.)	T98G and U-87 MG glioblastoma cells/induction of cell death, reduced proliferation and invasives; TB exclusion	[76]
β-elemene (*Curcuma wenyujin*)	β-elemene-derived antitumor drug/antitumor mechanisms and structural modification A375 melanoma cells/β-elemene enhances radio sensitization	[77,78]

**Table 3:** Examples of terpenoids with protective activities against CVDs reported in the literature over the last five years.

Terpenoid	Assay/Reported Activities	Ref.
Artemisinin	Rats/attenuates doxorubicin-induced cardiotoxicity and hepatotoxicity	[84]
	Rats/renoprotective effects on IgA nephropathy by suppressing NF-κB signaling and NLRP3 inflammasome activation by exosomes	[47]
	Human macrophage U937 cells/anti-inflammatory effect on uric acid-induced NLRP3 inflammasome activation through blocking the interaction between NLRP3 and NEK7	[85]
	I/R model rats/suppresses myocardial ischemia–reperfusion injury via NLRP3 inflammasome mechanism	[86]
Bakuchiol	C57BL6 male mice/protective effect limiting the synthesis of fibrosis, preventing oxidative damage and cell death in diabetic myocardium	[87]
	C57BL/6J mice and NRCM cells/antihypertrophy effects by modulating the synthesis of fibrosis and inflammatory responses	[88]
Betulin	Diabetic mice and glucose-stimulated H9c2 cells/protective impact on injured myocardium; significant reduction in cardiac inflammation (anticardiac inflammatory factor via the SIRT1/NLRP3/NF-κB pathway)	[89]
	I/R model rats/significantly improved the abnormal electrocardiograms; decreased myocardial infarction area and expression of myocardial enzymes and inflammatory cytokines and SITI1; decreased protein expression levels of NLRP3 and NF-κB (anti-inflammatory mechanism is associated with the NLRP3/NF-κB signaling pathway)	[90]
Carnosic acid	C57BL/6 mice/antiobesity effect by improving hormone homeostasis and reduced gene expression of liver lipogenesis possibly via the PPAR-γ pathway	[91]
	C57BL/6 mice/cardioprotective effect against myocardial remodeling by modulation oxidative stress and apoptosis via the AKT/GSK3β/NOX 4 signaling pathway	[92]
	C57BL/mice and H9c2 cells/protects the heart against toxicity via the suppression of oxidative damage, inflammation, apoptosis, and autophagy	[93]
Carnosol	H9c2 cells/protective effect against inflammation in the cardiomyoblasts may be via the NF-κB signaling pathway	[94]
	MAPC cells/promote vascular health by regulating redox status and downregulating inflammation and apoptosis	[95]
Carvacrol	Wistar rats/protective effect against myocardial hypertrophy by improving blood pressure and inhibiting apoptosis via regulation of the Bcl-2 family protein	[96]
Celastrol	Rat primary cardiomyocytes and H9C2 cells/prevents myocardium fibrosis and hypertrophy produced by angiotensin II	[97]
Hinokitiol	SEVC4-10 and A7r5 cells/protective effect against atherosclerosis by modulating cell adhesion molecules and members of the matrix metalloproteinase (MMP) family	[98]
	AC16 cells/protects cardiomyocytes from oxidative damage by regulating apoptosis and autophagy, probably through the GSK3β signaling pathway	[99]
Ferruginol	C57BL/mice and H9c2 cells/cardioprotective action by preserving the mitochondrial from the production of ROS, limiting damage to heart function, and attenuating the apoptotic process, possibly via the SIRT1 pathway that mediates mitochondrial biogenesis and fatty acid oxidation	[100]
	Wistar rats/cardioprotective effect against myocardial infarction via modulation of inflammatory response and upregulation of antioxidant enzymes	[101]
Geniposide	Spontaneous hypertensive rat/modulates blood pressure by inhibiting the WNK pathway mediated by the estrogen receptors	[102]
	I/R model rats and H9C2 cells/inhibition of autophagy via geniposide protects against myocardial I/R injury	[103]
	Neurons and PC-12 cells/inhibits NLRP3 inflammasome activation via autophagy in BV-2 microglial cells exposed to oxygen–glucose deprivation/reoxygenation	[104]
	Mice/protects against sepsis-induced myocardial dysfunction through AMPKα-dependent pathway	[105]
Oridonin	Renal proximal tubular epithelial cells and acute lung injury mice model/suppressed NF-κB or MAPK activation and release of TNF-α and IL-6	[106,107]
	Rats/drastically diminish the extent of myocardial infarction and the blood cardiac enzymes	[108]
	Mice/attenuates myocardial I/R injury by downregulating oxidative stress and the NLRP3 inflammasome pathway	[109]
	Mouse models of peritonitis, gouty arthritis, and type 2 diabetes/specific covalent inhibitor of NLRP3 inflammasomes, inhibiting the assembly and activation of NLRP3 inflammasomes	[110]
	RAW264.7 cells and mouse model/protects LPS-induced acute lung injury by modulating Nrf2-mediated oxidative stress and Nrf2-independent NLRP3 and NF-κB pathways	[111]
Pterostilbene	Rat heart subjected to ischemia–reperfusion/attenuates inflammation via the TLR4/NF-kB signaling pathway	[112]
Sweroside	H9c2 cells/protects against myocardial ischemia–reperfusion injury by inhibiting oxidative stress and pyroptosis partially via modulation of the keap1/Nrf2 axis	[113]
	H9c2 cells/ameliorate the cardiotoxicity of aconitine and the incidence of arrhythmias generated by aconitine	[114]
	H9c2 cells/protect against myocardial ischemia–reperfusion injury by inhibiting oxidative stress and pyroptosis partially via modulation of the keap1/Nrf2 axis.	[113]
	NASH mouse model/prevents non-alcoholic steatohepatitis by suppressing activation of the NLRP3 inflammasome	[115]
Thymol	Albino Wistar rats/cardioprotective effect against myocardial infarction by modulating oxidative stress, inflammation, and apoptosis	[116]
Triptolide	Mice and mouse cardiac fibroblasts/significantly inhibit the activation of NLRP3 inflammasome and show an antifibrosis effect	[117]

I/R—ischemia–reperfusion injury; NASH—non-alcoholic steatohepatitis.

## Data Availability

Not applicable.
